# Cost of individual peer counselling for the promotion of exclusive breastfeeding in Uganda

**DOI:** 10.1186/1478-7547-9-11

**Published:** 2011-06-29

**Authors:** Lumbwe Chola, Lungiswa Nkonki, Chipepo Kankasa, Jolly Nankunda, James Tumwine, Thorkild Tylleskar, Bjarne Robberstad

**Affiliations:** 1 Central Statistical Office, Box 31908, Lusaka, Zambia; 2 Centre for International Health, University of Bergen, Box 7804, N-5020 Bergen, Norway; 3 Health Systems Research Unit, Medical Research Council, Box 19070, Tygerberg, 7505, South Africa; 4 Department of Paediatric and Child Health, University of Zambia School of Medicine, Private Bag RXW1, Lusaka, Zambia; 5 Department of Paediatrics and Child Health, Makerere Medical School, Box 7072, Kampala, Uganda

## Abstract

**Background:**

Exclusive breastfeeding (EBF) for 6 months is the recommended form of infant feeding. Support of mothers through individual peer counselling has been proved to be effective in increasing exclusive breastfeeding prevalence. We present a costing study of an individual peer support intervention in Uganda, whose objective was to raise exclusive breastfeeding rates at 3 months of age.

**Methods:**

We costed the peer support intervention, which was offered to 406 breastfeeding mothers in Uganda. The average number of counselling visits was about 6 per woman. Annual financial and economic costs were collected in 2005-2008. Estimates were made of total project costs, average costs per mother counselled and average costs per peer counselling visit. Alternative intervention packages were explored in the sensitivity analysis. We also estimated the resources required to fund the scale up to district level, of a breastfeeding intervention programme within a public health sector model.

**Results:**

Annual project costs were estimated to be US$56,308. The largest cost component was peer supporter supervision, which accounted for over 50% of total project costs. The cost per mother counselled was US$139 and the cost per visit was US$26. The cost per week of EBF was estimated to be US$15 at 12 weeks post partum. We estimated that implementing an alternative package modelled on routine public health sector programmes can potentially reduce costs by over 60%. Based on the calculated average costs and annual births, scaling up modelled costs to district level would cost the public sector an additional US$1,813,000.

**Conclusion:**

Exclusive breastfeeding promotion in sub-Saharan Africa is feasible and can be implemented at a sustainable cost. The results of this study can be incorporated in cost effectiveness analyses of exclusive breastfeeding promotion programmes in sub-Saharan Africa.

## Background

Sub-Saharan Africa has the poorest child health record, accounting for over half of all deaths of children worldwide [1,2]. The most common causes of mortality are pneumonia and diarrhoea, together accounting for over 30% of child deaths [2,3], but these diseases may in part be prevented by exclusive breastfeeding [4]. Exclusive breastfeeding (EBF) of infants is, therefore, accepted as the most appropriate form of infant feeding [5,6]. Though the health benefits of EBF have been documented in various studies [7-9], this form of infant feeding is not universal, with about 40% of all children below 6 months exclusively breastfed worldwide in 2007 [10]. A study conducted in Mbale district in the eastern region of Uganda showed that though breastfeeding was common, exclusive breastfeeding was low, with only about 7% of children 3 months old fed exclusively on human milk [11].

EBF promotion has been identified as one of the interventions with the highest life-saving potential globally, and if all children were optimally breastfed, this could potentially save 13% of child deaths worldwide [12]. It is therefore unfortunate that the fear of breast milk transmitting HIV-I has led to EBF promotion largely being brought to a standstill in sub-Saharan Africa [13,14]. Advanced maternal HIV-I disease is associated with increased risk of transmission through breastfeeding [15]. However, the risk of HIV transmission was recently found to be lower with exclusive breastfeeding, compared to mixed feeding [16]. Mixed feeding also presents increased risk of children dying from causes such as diarrhoea in settings with unhygienic environments and unsafe feeding options [1]. The current recommendation for infant feeding is, therefore, that both women with unknown or negative HIV status and HIV infected women should also always be encouraged to exclusively breastfeed for six months post partum, unless replacement feeding is acceptable, feasible, affordable, sustainable and safe for both mother and child [6].

EBF promotion programmes are also hampered by many other factors, including cultural aspects at the family level and scarcity of resources at the national level [17-19]. Information on the effectiveness of methods to promote exclusive breastfeeding is available [16,20], but data on the costs of such programmes are scarce, particularly in sub-Saharan Africa. This hinders investment in public health programmes, and largely hampers priority setting, and may lead to the adoption of interventions that are less or not cost-effective [21]. There is, therefore, need to make this information available.

We set out to measure the costs of an individual peer counselling intervention, designed to increase EBF prevalence at 3 months postpartum among infants in sub-Saharan Africa [22]. PROMISE EBF was a multi-centre community randomised trial (http://clinicaltrials.gov/ct2/show/NCT00397150) conducted in four sub-Saharan African countries, namely Burkina Faso, South Africa, Uganda and Zambia. This paper presents the annual costs of the PROMISE EBF intervention in Uganda, and provides estimates of the resources required to fund the scale up to district level. The costing study was not done in Burkina Faso at the same time, due to the language insufficiency of the researchers, and both this and the South African report will be made at a later stage, albeit with slightly different focus. The Zambian study was not analysed due to disruptions caused by flooding at the time of the intervention.

### Study setting and intervention

Mbale district is situated in Eastern Uganda with a population of about 700,000 and a population density of 535 per square kilometre [23]. The study was carried out in two of the seven counties of the district: the urban Mbale municipality, situated approximately 230 km from the Ugandan capital, Kampala, and the rural Bungokho County. Mbale Municipality is the district centre and has approximately 10% of the district population [23]. Bungokho surrounds Mbale municipality and the population consists mainly of subsistence farmers. The majority are Bagisu who use Lumasaba as their main language, while some minority ethnic groups, Iteso, Baganda and Bagweri, speak different languages but are also able, most of the time, to understand Lumasaba.

In the district, 24 communities (clusters) were selected and stratified based on similarities in terms of location, urban-rural set ups and socio-economic status. In each stratum, half of the clusters were randomized to the control and intervention groups, respectively. The intervention of peer counselling for exclusive breastfeeding was therefore set up in twelve clusters, each with an estimated population of about 1000 inhabitants, expected to generate 35 babies in a year given a birth rate of 3.5%. Each rural cluster consisted of one to three villages combined, depending on the village population size. All pregnant women in the geographical clusters were eligible for participation. The women were identified in the clusters, introduced to the project and recruited upon consent, according to eligibility criteria. The inclusion criteria were that the woman resided in the selected cluster, was 7 months pregnant and had no intention to move out of the cluster. Women with severe psychological and somatic illness, those having given birth more than 1 week ago, and those planning to replacement feed were not included in the study.

### Peer counselling intervention

In the intervention clusters, mothers were visited by a peer supporter at least five times, with the first visit occurring when a mother was about seven months pregnant. The remaining visits were scheduled at 1, 4, 7 and 10 weeks after delivery. Mothers with breastfeeding problems were given extra visits. Extra visits were also given if a mother called the peer counsellor for additional assistance outside the scheduled time or if the peer counsellor deemed it necessary. The peer counsellors chose the time most convenient to the mothers for meetings during the scheduled weeks. The peer counselling intervention was conducted in a period of about one and a half years. Women in the control clusters did not receive this counselling, but were encouraged to attend regular antenatal and postnatal clinics, which are available at all health facilities in Uganda. Antenatal attendance is very high in Uganda, with over 94% of pregnant women making regular visits [24].

### Selection of peer supporters

Sensitization workshops and meetings aimed at introducing the project to community leaders were held prior to commencement of the trial. The community leaders facilitated the mobilisation of local women to work as peer counsellors on the project. Twelve women were selected as peer supporters, one in each cluster (table 1).

**Table 1:** PROMISE EBF intervention staff

Staff category	Number	Salary
Research coordinator	1	Full time
Peer supporter supervisor	2	Full time
Driver	1	Full time
Peer supporter	12	US$20 per month
Recruiter	12	US$20 per month

At the beginning of the programme, all peer supporters were given six days training based on the WHO breastfeeding counselling course [25]. During training, proper timing of counselling visits and the key messages to share with the mothers during different visits were emphasized. All peer counsellors completed the training and started supporting mothers in their villages with breastfeeding.

### Peer supporter supervision

A team consisting of two peer supporter supervisors and the study coordinator were responsible for overall peer supporter supervision. The role of the peer supporter supervisor was to provide advice and support to the peer counsellors. Supervision was done through on-site observation of counselling sessions, which were conducted every fortnight. Monthly meetings for all peer supporters were also held at the office. These meetings provided an opportunity to share field experiences and sort out any problems encountered by the counsellors.

### Project structure

PROMISE EBF composed of the following employees: a research coordinator, who was the overseer of the entire project, a data manager, in charge of the database, a data collectors' supervisor, whose job was to manage the data collectors, peer supporter supervisors who were responsible for the peer supporters, peer supporters, recruiters and a driver. During the PROMISE-EBF intervention, there was no clear distinction between the intervention and research component for workers such as the driver and research coordinator, as they worked on both activities. In the costing study, a distinction was made between project evaluation and the intervention. Only costs of the intervention are included in this paper. The intervention was basically the peer support program, and project evaluation consisted of the data collection process. Since the cost analysis was done in retrospect, there was no way to measure the time spent on different activities by workers who belonged both to the intervention and evaluation. The project personnel were, therefore, divided between project evaluation and intervention using percent effort, which was determined through interviews with project staff:

Project evaluation: Research coordinator (60%), data manager (100%), data collectors' supervisor (100%), data collectors (100%), recruiters (50%), driver (60%).

Intervention: Research coordinator (40%), peer supporter supervisors (100%), peer supporters (100%), recruiters (50%), driver (40%).

### Remuneration

The research coordinator, peer supporter supervisors and driver were permanent staff on the project, and were therefore offered competitive salary packages. Peer supporters and recruiters were not permanent members of staff. They were offered a token US$20 every month for their participation (table 1), a figure that was arrived at in a meeting with peer supporters, where they agreed on this as adequate compensation for their time. The figure was also intended to amount to about 10% of an average school teacher's salary, taking into consideration the effort that peer supporters were expected to put into work.

## Methods

### Costing

Costing was undertaken from a local provider's perspective and involved the identification of all project costs relating to the intervention in the project's books of accounts and administrative records. Costs to the family and society at large are not included in this analysis. Costing was done across 5 major categories which were identified as the main activities of the peer support intervention. These were start-up, overheads, training, peer support and peer support supervision. The start-up category included all preparatory activity costs such as manual adaptation, training and workshops. The initial training of peer supporters for the intervention was included as a capital cost in start-up costs. All post-start up training of peer supporters was included as a recurrent cost in the category we refer to as Training. This includes all re-training of counsellors, and other trainings which may or may not have been related to the intervention. Overheads included items such as rentals, telephone and internet. Peer support included all items related to peer counselling, such as personnel and travel; and peer supervision included personnel, transport, equipment and costs of all activities related to overall project supervision.

Data collection was based on the Costing Guidelines for HIV Prevention Strategies [26]. Resource use and cost data were collected for the period December 2005 to June 2008, to include all costs incurred during the preparatory stage. All costs were adjusted to 2007 prices using a Consumer Price Index (CPI) [27]. All prices were collected in local currency (Uganda Shillings, UGX) and converted to United States Dollars (US$) at an average exchange rate of UGX1,800 to US$1 [27]. Both financial and economic costs were calculated, where financial costs were the actual expenditures incurred in the purchase of items, and economic costs included the opportunity cost of resource use. Costs were classified as capital or recurrent costs. Recurrent costs included items such as stationery, fuel, utilities and personnel time. Capital costs included items such as vehicles, computers and furniture, and other items whose useful life was more than a year. Items whose useful life was more than a year but cost US$100 or less were classified as recurrent costs.

Capital costs were annuitized, a process that is used to reflect their annual value [28]. Start up costs were treated as capital and therefore annuitized. The annual financial cost of capital items was calculated using a straight line depreciation method, where the total cost of an item was divided by its useful life years. The annual economic cost of capital items was calculated using a discount rate of 20% [27]. We used useful life years of 3 years for start-up costs, 2 years for bicycles, 7 years for motor vehicles, 7 years for furniture, and 5 to 6 years for office equipment [29,30].

### Time use

We conducted a time use assessment among peer supporters to enable us to understand how time was spent on project activities. We specifically sought to find out how much time was spent per visit. A daily log sheet was administered to peer supporters, where they were asked to record the time spent on each counselling visit and the time it took to travel to each counselling session. We did not have a defined sample of counselling sessions, but decided to collect data for at least 500 visits. Total time spent on all activities was divided by the total number of activities, to calculate the average time spent on each activity. Time data were also used to calculate total and average walking distance covered by peer supporters for every visit, based on an estimated average walking speed of 5 kilometres (km) per hour (h).

### Outputs and average costs

The impact of the intervention was measured using the number of women counselled (which was taken as the number of women reached by the intervention) and exclusive breastfeeding up to 6 months post partum. The two main outputs of the peer support component from the costing perspective were total number of mothers counselled and total number of counselling sessions or visits. These were combined with total costs to calculate average costs per visit and per mother counselled. We used these to approximate the cost per weeks of exclusive breastfeeding (WEBF). WEBF were the sum of the duration, in weeks, that a child was exclusively breastfed. The cost per WEBF was expressed as the total cost divided by total WEBF at a given period.

### Sensitivity analysis

A univariate sensitivity analysis was undertaken to estimate the impact of a number of assumptions made in the analysis. The discount rate was varied by replacing the bond rate (20%) with 3% and 6%, which are both commonly used in health economic evaluations. We varied personnel costs up and down by 20%. We also estimated the impact of increasing or reducing the number of visits per mother (+/- 20%) and allocating staff time between project implementation and evaluation (+/-20%), for the project coordinator and driver, who were involved in both activities. In addition, we varied the percentage allocation of other costs shared between project implementation and evaluation (+/-20%). The +/-20% range was used because we did not have any reference studies or procedures, and we felt this range would capture as much variation as possible.

### Estimating the costs of an alternative community EBF promotion programme

PROMISE EBF was held in a trial setting, and as such, its design was oriented towards research. A number of activities and costs might not have been incurred in a programme setting, and total project costs might therefore be lower. We tested this assertion by modelling the costs of undertaking an alternative exclusive breastfeeding support programme at the scale of PROMISE EBF. We assumed that the community programme was supervised by the Ministry of Health (MOH), and undertaken through its network of community health workers such as traditional birth attendants and other voluntary workers, already established in most communities in Uganda. The aim was to assess the changes in costs and average costs of undertaking similar EBF support programmes with varying scenarios. The design of the alternative programme was based on a community randomized trial assessing the effects of community based promotion of exclusive breast feeding [20]. In the alternative programme therefore, we estimated the additional cost of adding a breastfeeding intervention programme to routine public sector programmes. We examined the costs of providing community support for EBF at the same level of intensity as PROMISE EBF. The expected numbers of births were the same in the alternative programme as in PROMISE EBF, therefore, the ratio of babies to counsellors was the same. In this alternative setup, we maintained the start-up activities and costs, as these are unlikely to change between trial and programme setting (table 2). The only overhead cost included was communication, which as in PROMISE, catered mostly for mobile telephone time for peer supporter supervisors. We excluded the specialised personnel, but maintained two peer supporter supervisors and peer supporters and recruiters at the same cost as PROMISE EBF. We also maintained the same number and cost of peer supervisory visits and meetings. Transport costs included fuel costs and other travel by the programme team.

**Table 2:** Inputs included in the major cost categories, PROMISE EBF and alternative scenario

Cost categories	PROMISE EBF	Alternative scenario
**Start up**	• Useful life - 3 years	• Useful life - 3 years
**Overheads**	• Utilities • Rentals • Communication	• Communication
**Peer support**	• Peer supporter's allowance • Field materials (bags, raincoats, stationery)	• Peer supporter's allowance • Field materials (bags, raincoats, stationery)
**Peer supervision**	• Personnel cost (Driver, Peer supervisors, Coordinator) • Transport costs (fuel, vehicle maintenance, insurance) • Supervisory visits and meetings • Office supplies • Office equipment and furniture • Motor vehicle	• Personnel cost (peer supervisors) • Transport costs (fuel, other transport) • Supervisory visits and meetings • Office supplies

### Scaling up EBF promotion to district level

We estimated the cost of scaling up the alternative scenario from the village level to district level, based on the calculated average costs and the expected number of annual births. The number of births was calculated based on an expected 35 babies born per thousand populations per year. We assumed that the cost structures observed in the sample were similar to those obtaining at district level.

## Results

The results of the PROMISE EBF project show that the intervention was successful in increasing EBF prevalence. At 12 weeks of age, based on a 24 hour recall, the results in the intervention and control arms were: 81.6% and 43.9% (PR 1.89; 95%CI 1.70-2.11). Similarly, at 24 weeks of age, the results were 58.6% and 15.5% (PR 3.83; 95%CI 2.97-4.95). The 7 day recall prevalence ratios were similar to those obtained in the 24 hour recall. The full analysis and discussion of the intervention methods and results are presented elsewhere [31].

### Costs

Table 3 presents the total project economic costs by input categories. Total costs amounted to US$56,308, with peer supervision accounting for the largest proportion (53%). This was followed by peer support with 26%, start-up costs with 13% and overhead costs with 8%. In the largest cost contributor, peer supervision, the major cost driver was personnel cost, which accounted for 48%. Transport costs accounted for 38% of total supervision costs.

**Table 3:** Total project costs (US$)

	Costs	% within inputs	% of total cost
			
**Startup**	**7 548**	**100%**	**13%**
Travel	5 188	69%	
Manual adaptation and initial training	2 360	31%	
			
**Overheads**	**4 345**	**100%**	**8%**
Communication	2 245	52%	
Utilities	226	5%	
Office rent	1 874	43%	
			
**Peer support**	**14 495**	**100%**	**26%**
Personnel cost	13 999	97%	
Bicycles	-	-	
Field materials	496	3%	
			
**Peer supervision**	**29 920**	**100%**	**53%**
Personnel cost	14 236	48%	
Transport costs	11 271	38%	
Supervisory meetings	317	1%	
Office Supplies	1 885	6%	
Capital costs	2 212	7%	
			
**Total**	**56 308**		**100%**

### Peer counsellors' time use

Counselling and travelling time data were collected for a sample of 1,192 visits. The total project time recorded was 184,786 minutes. Over 60% (120,573) of this time was spent travelling. The mean travel time in a day was 101 minutes, ranging from a low of 3 minutes to a high of 335 minutes. A total of 64,213 minutes were recorded for counselling sessions, with an average of about 54 minutes (range; 4-180 minutes) per counselling session. The mean distance walked per day per peer supporter was 8 km (range; 0-28 km).

### Outputs and average costs

A total number of 406 women were recruited into the PROMISE EBF intervention arm (table 4). The ratio of peer supporters to project participants was 1 to 34. We recorded a total number of 2,176 visits made by peer counsellors, which represents an average of about 5.4 visits per mother counselled, and 181 visits per peer counsellor. The average cost per counselling visit was US$26, and the cost per mother was US$139. The WEBF were estimated to be 3,876 at 12 weeks; and 5,568 at 24 weeks, based on the 24 hour recall of feeding practice [31]. The costs per WEBF were, therefore, US$15 at 12 weeks and US$10 at 24 weeks.

**Table 4:** Outputs and average costs

Outputs (number)	
Total women counselled	406
Total number of peer supporters	12
Total individual counselling visits	2176
Visits per woman	5.4
Visits per peer supporter	181
**Average costs (US$)**	
Total cost per individual counselling visit	26
Total cost per mother counselled	139

### Sensitivity analysis

The results of the sensitivity analysis are shown in table 5. The allocation of shared costs between PROMISE EBF research and peer counselling intervention had the greatest impact, increasing total costs by about 40%. Variation of the discount rate had a small impact on the costs, reducing costs by less than 10. Reducing the percentage of salaries paid to personnel also led to a decrease in costs of about 20%.

**Table 5:** Sensitivity analysis

	Total costs	Cost per visit	Cost per mother
**Baseline value (US$)**	**56,308**	**26**	**139**
Salary +20%	16%	8%	8%
Salary -20%	-19%	-11%	-11%
Discount rate 3%	-6%	-6%	-6%
Discount rate 6%	-5%	-5%	-5%
Visits +20%		-15%	
Visits -20%		24%	
Shared costs+20%	40%	30%	30%
Shared costs-20%	-40%	-30%	-30%

### Estimating the costs of an alternative community EBF promotion programme

The comparative economic costs of PROMISE EBF and the alternative scenario are presented in table 6. The costs of the community based programme were about 80% lower than PROMISE total costs. The cost per visit reduced to US$14 and the cost per mother reduced from US$139 to US$74.

**Table 6:** Comparative economic costs of PROMISE EBF and an alternative scenario

Cost categories	PROMISEEBF	Alternative
	(US$)	(US$)
Startup	7 548	7 548
Overheads	4 345	643
Peer support	14 495	14 495
Peer supervision	29 920	7 879
		
Total	56 308	30 365

		
**Average costs**		
Cost per visit	26	14
Cost per mother	139	74

### Scaling up EBF promotion to district level

Given that the total population of Mbale is 700,000, an annual birth rate of 35 per 1000 population, yields a total of 24,500 [(700,000 × 35)/1000] babies born per year in the district. We established that the average cost per mother-baby pair of the community intervention was US$74. Multiplying the average cost to the estimated number of babies yields an annual cost of US$1,813,000 (24,500 × 74). This is the estimated annual cost of implementing a breastfeeding intervention programme as an additional cost to routine public sector programmes. The total cost of scaling up the programme to a population of 1 million inhabitants was US$2,590,000.

## Discussion

We have analysed the costs of implementing an individual peer counselling intervention, which was successful in increasing exclusive breastfeeding prevalence at 3 months post partum [31]. A literature search for similar costing studies undertaken in sub-Saharan Africa yielded only one such study [32]. To our knowledge, this is the first detailed costing study of a breastfeeding intervention programme in Uganda. We present the trial intervention costs, and also estimate the additional costs of implementing such a project as part of existing public sector health programmes. An attempt was also made to estimate the annual costs of implementing the programme at district level.

PROMISE EBF was a moderately intensive study, but with very close supervision, and as such, personnel costs accounted for the largest share of total costs. Personnel costs were also driven up because permanent project staff were offered competitive salaries, equivalent to those prevailing in the private sector. Transport costs, and mostly fuel, also accounted for quite a large proportion of total costs. This was probably a result of the large distances that had to be covered during each visit, as evidenced by the results of the time use analysis, which indicated an average walking distance of 8 kilometres a day per peer supporter.

At US$139 per mother, PROMISE EBF was quite an expensive undertaking, whose design would be difficult to implement in Uganda given the scarcity of resources. However, our sensitivity analysis indicates that it is possible to reduce these costs by over 70%, through variation of certain component costs. Since PROMISE EBF was vertically conducted in a trial setting, the costs incurred might not be reflective of actual costs that a similar project would attract in a real life setting. We explored this assumption by assuming a horizontal project undertaken under the auspices of the Ministry of Health, with its network of community health workers that are well established in most communities in Uganda. This analysis revealed that it was possible to reduce annual implementation costs by over 60%. This reduction is possible for a number of reasons, most important that supervision is not as rigorous in real life as it is under trial settings. Trial supervision accounted for the highest cost in PROMISE EBF. Costs such as personnel emoluments in a trial setting are usually pegged at competitive market prices, but in a real setting, and under a government programme, these costs are substantially reduced as government workers are often paid a much lower rate. Sustainability of the project in the long term by the community, particularly through government support might therefore be feasible, with government personnel taking on a supervisory role.

Reducing costs as such, may be fairly easy. The real challenge lies in simultaneously maintaining the quality of intervention outcomes. The effect of cost reductions on the quality of outcomes has not been analysed in this paper, but it is necessary for future research to look into this issue.

The benefits of exclusive breastfeeding have been documented [4,5], and there is no doubt that it is important to implement breastfeeding promotion programmes in low income countries. It is essential, however, to identify appropriate programmes that can be implemented at minimum cost, as we have attempted to do by suggesting an alternative programme to PROMISE EBF, which can be implemented as part of existing public sector programmes. Scaling up such a programme to district level would cost an estimated US$1,813,000 in additional annual health expenditures to Mbale district, or US$2,590,000 per 1 million inhabitants in Uganda. We compared these costs to those obtained in a costing study of the PROMISE EBF in Zambia [Chola L et al; *Costs of a peer counselling for the promotion of exclusive breastfeeding in Zambia*; Unpublished], and found that while average costs were lower in Uganda, the scale up costs were much higher. The scale up costs at district level were estimated at about US$700,000, obviously a result of population differentials, with Mbale district in Uganda having a higher population. The cost per mother of US$139 in Uganda, compared to US$233 in Zambia indicated higher cost structures in the latter country, highlighting the need for policy makers to take the economic environment into consideration when planning such programmes. Both the Zambian and Ugandan costs, however, were comparatively lower than similar costs estimated for implementing such a programme in Kwazulu Natal, South Africa [32]. Horton et al estimated an average of about US$7 per child to scale up a behaviour change intervention such as PROMISE EBF [33]. This estimate was made at a regional level, for 36 low income countries in Asia, sub-Saharan Africa, Latin America and Europe, and thus may not compare well with our estimate which was programme and country specific. Cost comparisons are usually difficult to make across countries, as the cost structures may differ greatly. It is also difficult to make comparisons between our estimate and Horton's due to the limited information given on the assumptions made in the Horton study.

Whether our estimated costs of scaling up the programme are attainable, or not, by the governments in our study areas is debatable, but it is important to acknowledge the availability of external funding sources, and that implementing such a programme on a large scale will require concerted efforts by the government and its partners in the donor community, private sector and non-governmental organisations. All parties involved in the implementation of such an intervention, should however, be aware of the challenges of implementing it both as a vertical or integrated programme. Implementing such an intervention as part of a government programme for example, could have a negative impact on the performance of an already overstretched workforce. Such project effects should be known before hand and solutions made to mitigate them.

Investments do not depend on cost decisions alone, and it is prudent to weigh costs against the benefits of an intervention before making an investment. In the case of PROMISE EBF or similar breastfeeding support programmes, policy makers may need to reflect on the potential long term health and economic benefits that could arise from the promotion and maintenance of exclusive breastfeeding through peer support. Evidence confirms that it is possible to reduce costs of illness due to diseases such as diarrhoea and pneumonia, by increasing the level of exclusive breastfeeding, thereby accruing savings from reduced service provision [34-37]. While these studies were not undertaken in the sub-Saharan African context, their evidence should be treated as important to reflecting the possibilities of attaining such economic benefits of exclusive breastfeeding. These added values of breastfeeding should be incorporated in a full economic evaluation of exclusive breastfeeding promotion in sub-Saharan Africa to provide a fuller picture of health benefits as well as costs. This should ideally be undertaken from a societal perspective, to try and capture the wider range of costs that this current analysis omitted. We restricted our analysis to health provider costs, thereby potentially underestimating the true cost of peer counselling for exclusive breastfeeding.

Future research should also look into the interaction of costs and time spent on the project. We did not analyse this relationship, though we conducted a time use survey among some project staff. It can be envisaged though, that the more time spent, the higher would be the costs. How this would affect the quality of counselling is debateable, but it can be argued that the effect would most likely be negative, as peer supporters might concentrate on increasing the number of visits in an effort to increase their monetary gain, rather than spending more time on each visit to ensure high quality support.

## Competing interests

The authors declare that they have no competing interests.

## Authors' contributions

All authors participated in the design of the study. LC, LN and BR contributed to data analysis, writing and editing of the manuscript. CK, JN, JT and TT contributed to the writing and editing of the manuscript. LC made the first draft of the manuscript. All authors read and approved the final manuscript.
